# Impact of Sugar Tax on Sugar-sweetened Beverage Consumption among Saudi Schoolchildren

**DOI:** 10.3290/j.ohpd.b1075081

**Published:** 2021-03-17

**Authors:** Muhanad Alhareky, Sumit Bedi, Abdullah AlMulhim, Maha El Tantawi, Faraz A. Farooqi, Jehan AlHumaid

**Affiliations:** a Assistant Professor, Department of Preventive Dental Sciences, College of Dentistry, Imam AbdulRahman bin Faisal University, Dammam, Saudi Arabia. Wrote manuscript, collected/analysed data, contributed to discussion, final review.; b Lecturer, Department of Preventive Dental Sciences, College of Dentistry, Imam AbdulRahman bin Faisal University, Dammam, Saudi Arabia. Wrote manuscript, collected/analysed data, contributed to discussion, final review.; c Teaching Assistant, Department of Preventive Dental Sciences, College of Dentistry, Imam AbdulRahman bin Faisal University, Dammam, Saudi Arabia. Wrote manuscript, collected/analysed data, contributed to discussion.; d Professor, Department of Pediatric Dentistry and Dental Public Health, Faculty of Dentistry, Alexandria University, Alexandria, Egypt. Performed statistical analysis, contributed to discussion.; e Lecturer, College of Dentistry, Imam AbdulRahman Bin Faisal University, Dammam, Saudi Arabia. Performed statistical analysis, contributed to discussion.; f Associate Professor, Department of Preventive Dental Sciences, College of Dentistry, Imam AbdulRahman Bin Faisal University, Dammam, Saudi Arabia. Manuscript writing contributed to discussion, final review.

**Keywords:** energy drinks, sugar consumption, sugar-sweetened beverages, sugar tax

## Abstract

**Purpose:**

In 2017, Saudi Arabia introduced a 120% tax on energy drinks and a 50% tax on soft drinks. The impact of this policy on the consumption of sugar-sweetened beverages (SSB) among schoolchildren is not known in this country. The present study evaluated the impact of the excise tax on SSB consumption in the tri-city metropolitan area of Dammam-Khobar-Dhahran, Eastern Province, Saudi Arabia.

**Materials and Methods:**

A repeated cross-sectional design was used to examine the difference between pre- and post-tax SSBs consumption among schoolchildren (12–14 years old) in Dammam-Khobar-Dhahran cities. A beverage-consumption frequency questionnaire was completed by 453 participants before the tax implementation and 334 participants after the tax implementation. The tax on soft drinks was increased by 50% and on energy drinks by 120%. Pre-tax data were collected in May 2017 and post-tax data in April 2018.

**Results:**

The proportion of participants who consumed energy drinks was 46.1% (95% CI: 42-51) before tax implementation, decreasing to 38.4% (95% CI: 33-44) after tax implementation, a reduction of nearly 8%. 92.5% (95% CI: 90-95) of the participants consumed soft drinks before tax implementation and 94.6% (95% CI: 92-97) did so after tax implementation, an increase of about 2%.

**Conclusions:**

The study showed no statistically significant impact of tax implementation on the consumption of energy drinks and soft drinks in this sample of children.

Excess sugar intake is a key causative factor for obesity, type 2 diabetes, and dental caries.^1,21^ Sugar-sweetened beverages (SSB) are the chief source of added sugars, due to wide availability and aggressive marketing. They have also become more affordable, leading to increased consumption in recent decades. SSB consist of many added sugars, which may detrimentally affect health due to frequent consumption over a period of time, despite their limited nutritional value.^2^ To prevent sugar-related diseases, the World Health Organization (WHO) has recommended that sugar consumed should not surpass 10% of total daily total calories and preferably make up ≤5% of total energy ingestion.^13^

A study from the US reported that added sugar intake increases with age and ranges from 3.5 g/day for children under 1 year of age to 102.1 g/day for 18-year-olds.^7^ Substantially less sugar is ingested by children under 6 years old than by children 6 to 18 years old. Alsubaie et al^2^ reported that 56.3% of 7- to 12-year-old Saudi children consumed carbonated beverages weekly and 17.1% daily. The consumption of added sugars increases from low-income families to middle-income families, then declines among children from the highest-income families.^13^

Adverse effects of SSB on oral and general health have been well documented in the literature, and intervention such as the sugar tax has been levied in many countries to reduce SSB consumption.^1,12,22^ In addition to widespread public health recommendations, public policies have been proposed to change consumption patterns at the population level. The most common actions implemented to reduce SSB consumption include taxation, reduction of availability in schools, restrictions on marketing to children, public awareness campaigns, and front-of-package labeling.^4,17,18,23^

Saudi Arabia implemented the excise tax on beverages such as soft drinks and energy drinks in June 2017 to control rising health issues related to SSB. Therefore, it was important to understand the impact of the policy on the consumption of SSB among schoolchildren. The study aimed to compare the consumption of SSB among schoolchildren in the Eastern province of Saudi Arabia before and after the implementation of the tax.

## Materials and Methods

A repeated cross-sectional study was performed to examine the changes in pre- and post-tax beverage consumption among schoolchildren (12–14 years) in Greater Dammam, Eastern Province of Saudi Arabia. Greater Dammam is the metropolitan area that consists of Dammam, Al Khobar, and Dhahran cities. The sample size calculation was based on population size (N ≈ 30,000), % frequency of outcome in the population (70%), confidence limits (4%), and design effect (1). The sample for pre-tax (N = 492) and post-tax (N = 492) data collection was calculated.

Six public schools were selected using a simple random sampling technique by employing the Excel 2010 (Microsoft; Redmond, WA, USA) random number generator. After the selection of schools, permission to visit and conduct the study was granted by the Ministry of Education, and schools were approached. In each school, classes and students were similarly selected using simple random sampling. Children who provided their written informed consent from their parents/legal guardians and expressed their willingness to participate were included in the study. Written consent included the purpose, benefits, and procedure of the study. The contact details of the principal investigator were provided in case further information about the study was required by the parents/legal guardians. Ethical approval was obtained from an institutional review board at Imam Abdulrahman Bin Faisal University.

Saudi Arabia announced the tax on SSB in May 2017, and it was implemented in June 2017. Tax implementation increased the prices of soft drinks (such as Pepsi, Coca Cola, Sprite, Fanta, 7up, etc.) by 50% and that of energy drinks (Red Bull, Bison, etc.) by 120%. Data were collected in two phases: pre-tax phase in May 2017 and post-tax phase in April 2018.

The questionnaire included information regarding nationality, socioeconomic background (monthly family income, education levels of parents, employment status of parents), and daily consumption of SSB products (soft drinks and energy drinks). Participants who consumed one or more can/bottle of SSB per day were defined as daily users. The questionnaire was administered among 492 participants during the pre-tax phase. Similarly, 492 participants were approached during the post-tax phase at the same schools 11 months after tax implementation. The participants were provided with a self-administered questionnaire in paper form in their classrooms. During questionnaire administration, participants’ queries were addressed by the study investigators, and completed questionnaires were collected within 15–20 minutes in each classroom. Nearly one month was spent on data collection in each phase, pre- and post-tax. At the times of data collection, there were no restrictions on the availability of SSB at the schools.

Data were gathered initially in MS Excel (2016) and statistical analysis was done using Statistical Package for Social Science (SPSS) Statistics for Windows, version 22 (IBM; Armonk, NY, USA). Descriptive statistics for demographic information (nationality, educational background, family income, and employment status) and the consumption of SSB before and after tax implementation were presented as frequencies and proportions with 95% confidence intervals. Pearson’s chi-squared test was performed to compare the proportions of different categories of participants who consumed SSB before and after-tax implementation. Univariate logistic regression analysis was conducted to evaluate the influence of demographic data on the consumption of SSB after tax implementation, and odds ratios and 95% confidence intervals were calculated. p < 0.05 was considered statistically significant.

## Results

A total of 787 school children were included in the study. Of 453 participants before tax implementation, 354 (78%) were Saudi and 99 (22%) were non-Saudi. After tax implementation, the study included 288 (87%) Saudi and 46 (13%) non-Saudi participants. Most participants had college/university educated parents: 63% before tax implementation and 80% after tax implementation. The majority of participants had parents working in the private sector and belonged to high income group (over 20,000 SAR) before and after tax implementation (Table 1).

**Table 1:** Demographic characteristics of schoolchildren

Demographic variables	Before tax implementation N = 453 N (%)	After tax implementation N = 334 N (%)
**Nationality** Saudi Non-Saudi	354 (78)	288 (87)
	99 (22)	46 (13)
**Parents’ education** Not educated Primary/elementary school Highschool/diploma College/university	5 (1)	0 (0)
	29 (6)	5 (2)
	132 (29)	61 (18)
	287 (63)	268 (80)
**Parents’ employment** Unemployed Private job Government job	35 (8)	30 (9)
	344 (76)	213 (64)
	74 (16)	91 (27)
**Monthly family income** Less than 5000 SAR* 5000 to 10,000 SAR 10,000 to 15,000 SAR Above 20,000 SAR	32 (7)	6 (2)
	99 (22)	49 (15)
	159 (35)	111 (33)
	163 (36)	168 (50)

*1000 SAR is equivalent to 267 US$.

The proportion of soft drink consumption was 92.5% (95% CI: 90-95) before tax implementation and 94.6% (95% CI: 92-97) after tax implementation, an increase of about 2%. A significantly higher proportion of Saudi (72%, 95% CI: 68-77) than non-Saudi children (20.5%; 95% CI: 16-24) consumed soft drinks before tax implementation (p = 0.019). Similarly, after tax implementation, more Saudi (81.7%, 95% CI: 78-86) than non-Saudi children (12.9, 95% CI: 9-16) used soft drinks (p = 0.022). The study found that soft drink consumption before and after tax implementation was not statistically significantly associated with parents’ education, parents’ employment, and family income (Table 2).

**Table 2:** Comparison of soft drink consumption among schoolchildren before and after tax implementation

Demographic variables	Soft drink consumption			
	Before-tax proportions in % (95% CI)	p-value	After-tax proportions in % (95% CI)	p-value
**Nationality** Saudi Non-Saudi	72 (68-77)	0.019	81.7 (78-86)	0.022
	20.5 (16-24)		12.9 (9-16)	
**Parents’ education** Not educated Primary/elementary school Highschool/diploma College/university	1.1 (0-2)	0.617	9 (6-12)	0.805
	5.96 (4-8)		3.9 (2-6)	
	26.26 (22-30)		22.8 (18-27)	
	59.16 (55-64)		67.1 (62-72)	
**Parents’ employment** Unemployed Private job Government job	6.84 (64-73)	0.535	8.1 (5-11)	0.568
	71.08 (67-75)		65.3 (60-70)	
	14.5 (11-18)		21.3 (17-26)	
**Family income** Less than 5000 SAR 5000 to 10,000 SAR 10,000 to 15,000 SAR Above 20,000 SAR	6.8 (4-9)	0.982	1.8 (0-3)	0.716
	19.6 (16-23)		14.1 (10-18)	
	21.56 (27-36)		30.8 (26-36)	
	33.11 (29-37)		47.9 (43-53)	
Overall consumption	92.5% (90-95)		94.6% (92-97)	

Pearson’s chi-squared test was performed to compare the proportions (%) of different categories of participants before and after tax implementation (statistical significance set at p < 0.05).

The percentage of participants who consumed energy drinks was 46.1% (95% CI: 42-51) before tax implementation, decreasing to 38.4% (95% CI: 33-44) after tax implementation, a reduction of ca 8%. Only nationality was statistically significantly associated with energy drink consumption before and after tax implementation. A statistically significantly higher proportion of Saudi (37.96%, 95% CI: 33-42) compared with non-Saudi children (8.1%, 95% CI: 6-11) used energy drinks before tax implementation (p = 0.038). Similarly, statistically significantly more Saudi (34.2%, 95% CI: 29-39) vs non-Saudi children (4.2%, 95% CI: 2-6) consumed energy drinks after tax implementation (p = 0.028).

However, the study found no statistically significant associations of parents’ education, parents’ employment, or family income with the consumption of energy drinks after tax implementation (Table 3).

**Table 3:** Comparison of energy drink consumption among schoolchildren before and after tax implementation

Demographic variables	Energy drink consumption			
	Before-tax proportions in % (95% CI)	p-value	After-tax proportions in % (95% CI)	p-value
**Nationality** Saudi Non-Saudi	37.96 (33-42)	0.038	34.2 (29-39)	0.028
	8.1 (6-11)		4.2 (2-6)	
**Parents’ education** Not educated Primary/elementary school Highschool/diploma College/University	0.4 (0-1)	0.897	0 (0-0)	0.33
	3.33 (2-5)		0.3 (1-5)	
	14.56 (11-18)		8.4 (5-11)	
	27.81 (24-32)		29.6 (25-34)	
**Parents’ employment** Not working Private job Government job	2.6 (1-4)	0.058	3 (1-5)	0.407
	33.55 (29-38)		23.4 (19-28)	
	9.93 (7-13)		12 (9-15)	
**Family income** Less than 5000 5000 to 10,000 10,000 to 15,000 Above 20,000	3.3 (2-5)	0.420	0.6 (0-1)	0.835
	9.7 (7-12)		6.3 (4-9)	
	17.6 (14-21)		13.2 (10-17)	
	15 (12-18)		18.3 (14-22)	
Overall consumption	46.1% (42-51)		38.4% (33-44)	

Pearson’s chi-squared test was performed to compare the proportions (in %) of different categories of participants before and after tax implementation (statistical significance set at p < 0.05).

Table 4 shows the results of regression analysis of independent variables (nationality, parents’ education, parents’ employment, and family income) with the dependent variable (consumption of soft drinks and energy drinks after tax implementation). No statistically significant associations were observed between independent variables and the dependent variable in the study (Table 4).

**Table 4:** Univariate logistic regression analysis: association between demographic variables and soft drink/energy drink consumption after tax implementation

Dependent variables	Independent variables	OR (95% CI)
Soft drink consumption	**Nationality** Saudi Non-Saudi	1.55 (0.30-7.82)
	**Family income** High Middle/low	0.567 (0.14-2.67)
	**Education** Highschool or above Primary school education/no education	7.81 (0.94- 61.38)
	**Employment status** Private job Government job/no job	0.53 (0.06-4.54)
Energy drink consumption	**Nationality** Saudi Non-Saudi	1.49 (0.75-2.96)
	**Family income** High Middle/low	1.13 (0.19-6.55)
	**Education** Highschool or above Primary school education/no education	3.28 (0.34-31.25)
	**Working status** Private job Government job/no job	1.90 (0.77-4.77)

Univariate logistic regression analysis was conducted to evaluate the influence of demographic data on the consumption of SSB; 95% confidence interval was used.

## Discussion

The study showed reduced energy drink consumption and slightly increased soft drink consumption among schoolchildren aged 12-14 years after SSB tax implementation in Saudi Arabia. Our results reflect consumption changes in the metropolitan Greater Dammam region, comparable to the findings of other studies in the USA,^8,15,16,20,24^ Mexico,^9-11^ Chile,^3,4^ and France.^5^

In the first year after tax implementation, the present study found a reduction in energy drink consumption of 8% when the price increased by 120%. Falbe et al^14^ observed that a SSB price increase of 8% in Berkeley was associated with reduced consumption of approximately 10%. In Mexico, a 5.5% reduction in taxed beverage intake occurred in the first year, increasing to 9.7% in the second year; the reduction was greater in households with low socioeconomic vs high socioeconomic status (14.3% vs 5.6%, respectively).^11^

One would assume a substantial reduction in energy drink consumption after the implementation of a 120% tax in Saudi Arabia. However, there was an 8% decrease in the proportion of children who used energy drinks and a 2% increase in the proportion of children who consumed soft drinks after tax implementation in the present study. This shows that the implementation of tax on SSB alone is not sufficient to reduce the consumption among schoolchildren in Saudi Arabia. Other preventive measures, such as raising awareness about the adverse effects of SSB on health and restricting the availability of SSB in schools, are also needed.

Our results are corroborated by those of previous studies from the USA^5-7,20^ and Mexico,^8,9,11^ which may indicate that our tax-related 8% reduction in consumption of SSB may increase in near future. In their recent study in the Saudi population, Alsukait et al^3^ concluded that annual purchases of soda and energy drinks decreased by 41% and 58%, respectively, in 2018 (after the tax had been implementated) compared to 2016.

In the present study, a statistically significantly higher proportion of Saudi children compared to non-Saudi children consumed soft drinks and energy drinks before and after tax implementation. This reflects that the 120% increase in taxes on soft drinks and energy drinks differentially affected consumption among children of different nationalities in our study. Whether taxes on SSB to lower sugar and energy (calorie) consumption improve the standard of national diets and have useful effects on public health has yet to be scientifically evaluated. It is of equal importance to monitor changes in consumer behavior in response to SSB taxes as well as the response of the beverage industry to these challenges and consumer preferences. Ongoing evaluation of SSB taxes will be critical to determine the long-term impact.

### Limitations and Suggestions of the Study

The current study has certain limitations. Results from Greater Dammam, an urban area with a well-educated population, may not be applicable to other geographic areas due to lack of awareness and limited accessibility especially in remote areas. Therefore, the generalisability of the study findings should be considered cautiously when interpreting the results. Self-reported data are subject to bias due to under- and over-reporting of responses. The cross-sectional study design cannot be used to establish causality.

During the pre-tax phase, 453 participants provided their responses, whereas only 334 participants agreed to participate in post-tax phase, which is a dropout rate of 26.2%. Taxes were implemented in May 2017 and post-tax data were collected in April 2018. The impact of the tax on SSB consumption 11 months after implementation provided important information. However, immediate and long-term evaluations of tax implementation on SSB should be carried out in the future. The study provided a descriptive analysis of SSB consumption before and after tax implementation in different sociodemographic categories of schoolchildren. Therefore, influences of other factors such as physical activity, sedentary time, body mass index, other dietary factors, market share, and the existence of other programs on the consumption of SSB should be evaluated in large cohort studies.

The established relationship between SSB consumption and diseases such as obesity, diabetes, and dental caries is complex and requires wide-ranging solutions. Constant efforts are required to restrict the sale and purchase of SSB in schools. Early intervention plans can be tailored to different groups based on age, gender, language, and/or culture, with active participation of the local community. Taxation can reduce SSB consumption by direct economic incentives, earmarking revenues to support healthy foods, and sending a negative message. However, a higher tax rate may be necessary to have a measurable effect on decreasing the incidence of dental caries.

## Conclusion

The study showed no statistically significant impact of tax implementation on the consumption of energy drinks and soft drinks in this sample of children. Evaluating the impact of added-sugar excise taxes in different parts of kingdom will expand understanding of their public health benefits and their generalisability.

## References

[ref1] Afshin A, Peñalvo JL, Del Gobbo L, Silva J, Michaelson M, O‘Flaherty M (2017). The prospective impact of food pricing on improving dietary consumption: A systematic review and meta-analysis. PLoS One.

[ref2] Alsubaie ASR (2017). Consumption and correlates of sweet foods, carbonated beverages, and energy drinks among primary school children in Saudi Arabia. Saudi Med J.

[ref3] Alsukait R, Wilde P, Bleich S, Singh G, Folta S (2019). Impact of Saudi Arabia‘s sugary drink tax on prices and purchases. Curr Develop Nutrition.

[ref4] Briggs ADM, Mytton OT, Kehlbacher A, Tiffin R, Elhussein A, Rayner M (2017). Health impact assessment of the UK soft drinks industry levy: a comparative risk assessment modelling study. Lancet Public Health.

[ref5] Capacci S, Allais O, Bonnet C, Mazzocchi M (2019). The impact of the French soda tax on prices, purchases and tastes: an ex post evaluation. PLoS One.

[ref6] Caro JC, Corvalán C, Reyes M, Silva A, Popkin B, Taillie LS (2018). Chile‘s 2014 sugar-sweetened beverage tax and changes in prices and purchases of sugar-sweetened beverages: An observational study in an urban environment. PLoS Med.

[ref7] Chi DL, Scott JM (2019). Added sugar and dental caries in children: a scientific update and future steps. Dent Clin North Am.

[ref8] Colantuoni F, Rojas C (2015). The impact of soda sales taxes on consumption: evidence from scanner data. Contemp Econ Policy.

[ref9] Colchero MA, Guerrero-López CM, Molina M, Rivera JA (2016). Beverages Sales in Mexico before and after implementation of a sugar sweetened beverage tax. PLoS One.

[ref10] Colchero MA, Molina M, Guerrero-López CM (2017). After Mexico implemented a tax, purchases of sugar-sweetened beverages decreased and water increased: difference by place of residence, household composition, and income level. J Nutr.

[ref11] Colchero MA, Popkin BM, Rivera JA, Ng SW (2016). Beverage purchases from stores in Mexico under the excise tax on sugar sweetened beverages: observational study. BMJ.

[ref12] Dooley D, Moultrie NM, Sites E, Crawford PB (2017). Primary care interventions to reduce childhood obesity and sugar-sweetened beverage consumption: Food for thought for oral health professionals. J Public Health Dent.

[ref13] Eicher-Miller HA, Zhao Y (2018). Evidence for the age-specific relationship of food insecurity and key dietary outcomes among US children and adolescents. Nutr Res Rev.

[ref14] Falbe J, Rojas N, Grummon AH, Madsen KA (2015). Higher retail prices of sugar-sweetened beverages 3 months after implementation of an excise tax in Berkeley, California. Am J Public Health.

[ref15] Falbe J, Thompson HR, Becker CM, Rojas N, McCulloch CE, Madsen KA (2016). Impact of the Berkeley Excise Tax on sugar-sweetened beverage consumption. Am J Public Health.

[ref16] Fletcher JM, Frisvold DE, Tefft N (2015). Non-linear effects of soda taxes on consumption and weight outcomes. Health Econ.

[ref17] Lee JY, Giannobile WV (2016). Taxes on sugar-sweetened beverages: a strategy to reduce epidemics of diabetes, obesity, and dental caries?. J Dent Res.

[ref18] Muth ND, Dietz WH, Magge SN, Johnson RK (2019). Public policies to reduce sugary drink consumption in children and adolescents. Pediatrics.

[ref19] Nakamura R, Mirelman AJ, Cuadrado C, Silva-Illanes N, Dunstan J, Suhrcke M (2018). Evaluating the 2014 sugar-sweetened beverage tax in Chile: An observational study in urban areas. PLoS Med.

[ref20] Silver LD, Ng SW, Ryan-Ibarra S, Taillie LS, Induni M, Miles DR, Poti JM, Popkin BM (2017). Changes in prices, sales, consumer spending, and beverage consumption one year after a tax on sugar-sweetened beverages in Berkeley, California, US: A before-and-after study. PLoS Med.

[ref21] Tahmassebi JF, BaniHani A (2020). Impact of soft drinks to health and economy: a critical review. Eur Arch Paediatr Dent.

[ref22] Teng AM, Jones AC, Mizdrak A, Signal L, Genç M, Wilson N (2019). Impact of sugar-sweetened beverage taxes on purchases and dietary intake: systematic review and meta-analysis. Obes Rev.

[ref23] World Health Organization (2017). Taxes on sugary drinks: Why do it?.

[ref24] Zhong Y, Auchincloss AH, Lee BK, Kanter GP (2018). The short-term impacts of the Philadelphia beverage tax on beverage consumption. Am J Prev Med.

